# Paternal Preconception Metformin Use and Offspring Risk of Congenital Malformations

**DOI:** 10.1001/jamanetworkopen.2025.15002

**Published:** 2025-06-12

**Authors:** Krista F. Huybrechts, Loreen Straub, Ran S. Rotem, Brian T. Bateman, Sonia Hernández-Díaz

**Affiliations:** 1 Division of Pharmacoepidemiology and Pharmacoeconomics, Department of Medicine, Brigham and Women’s Hospital and Harvard Medical School, Boston, Massachusetts; 2 Department of Environmental Health, Harvard T.H. Chan School of Public Health, Boston, Massachusetts; 3 Kahn-Sagol-Maccabi Research and Innovation Institute, Maccabi Healthcare Services, Tel Aviv, Israel; 4 Department of Anesthesiology, Perioperative and Pain Medicine, Stanford University School of Medicine, Stanford, California; 5 Department of Epidemiology, Harvard T. H. Chan School of Public Health, Boston, Massachusetts

## Abstract

This cohort study examines whether paternal exposure to metformin during spermatogenesis is associated with major congenital malformations in children.

## Introduction

Metformin is widely used for glycemic control. A Danish cohort study raised concern that paternal preconception metformin use may increase risk of major congenital malformations (MCMs) in offspring, particularly genital defects in boys.^1^ Causal association was considered plausible due to metformin’s role in semen quality, but replication is necessary before this finding can guide clinical practice. We replicated the analyses in an independent cohort and expanded the design to disentangle associations with metformin use from associations with familial cardiometabolic conditions.

## Methods

The source cohort consisted of commercially insured mother-father linked pregnancies ending in a singleton livebirth from 2003 to 2020 (Merative MarketScan).^2^ The Brigham and Women’s Hospital Institutional Review Board approved this cohort study and waived informed consent because deidentified data were used. We followed the STROBE reporting guideline.

Two study designs were implemented. First, we closely replicated the study^1^ that initiated the safety signal. Fathers were considered exposed if they filled a metformin prescription during the 3 months before pregnancy (spermatogenesis period). Logistic regression was used to estimate the relative risk (RR) of MCM among fathers with metformin use vs no metformin use during spermatogenesis, adjusting for use before and after spermatogenesis, calendar year, parental age, and maternal smoking. Second, we restricted the cohort to fathers with type 2 diabetes and evaluated 6 exposure contrasts (Table) to discern potential confounding by underlying familial cardiometabolic risk factors using propensity score overlap weighting for confounding adjustment; see Table for covariates included in the propensity score.

**Table.** Risk of Major Congenital Malformations After Paternal Exposure to Metformin During Spermatogenesis Among Fathers With Type 2 Diabetes

Metformin use during spermatogenesis^a^	Relative risk (95% CI)	
	Unadjusted	Adjusted^b^
**Compared with no antidiabetics use^c^**		
Metformin monotherapy (n = 1254) vs no antidiabetic treatment (n = 1936)	1.64 (1.18-2.28)	1.52 (0.89-2.58)
Metformin monotherapy or combination therapy (n = 3234) vs no antidiabetic treatment (n = 1936)	1.32 (1.00-1.76)	1.22 (0.78-1.91)
**Compared with other antidiabetic treatments**		
Metformin monotherapy (n = 1254) vs other antidiabetic treatment (n = 932)	1.42 (0.96-2.11)	1.18 (0.67-2.10)
Metformin + insulin but no other antidiabetic treatment (n = 124) vs insulin monotherapy (n = 303)	0.98 (0.31-3.06)	1.15 (0.19-6.91)
**Compared with metformin use discontinuation** ^d^		
Metformin monotherapy continuation (n = 657) vs discontinuation (n = 261)	1.30 (0.67-2.52)	1.37 (0.52-3.61)
Metformin monotherapy or combination therapy continuation (n = 1889) vs discontinuation (n = 261**)**	1.11 (0.60-2.04)	1.06 (0.44-2.57)

^a^ Exposure contrasts: metformin or other antidiabetic treatment was assessed during spermatogenesis (last menstrual period [LMP] – 90 days to LMP).

^b^ Confounding adjustment: the cohort was restricted to (1) mothers without diabetes or hypertension and without use of cardiovascular medications and (2) fathers with a diagnosis of type 2 diabetes. Adjusted for calendar year and (proxies for or correlates of) cardiometabolic risk factors: maternal covariates (polycystic ovarian syndrome and infertility), maternal and paternal covariates (age, smoking, asthma, thyroid disease, hyperlipidemia, overweight or obesity, depression, anxiety, oral corticosteroids, thyroid replacement therapy, opioids, antidepressants, and antianxiety medication), and paternal covariates (alcohol use disorder or dependence, drug use disorder or dependence, hypertension, ischemic heart disease, congestive heart failure, arrythmia, kidney disease, β-blockers, diuretics, calcium channel blockers, angiotensin-converting enzyme inhibitors, and lipid-modifying agents).

^c^ Exposure contrasts: for fathers without antidiabetic treatment, no antidiabetic prescription fills were allowed in the 90 days before spermatogenesis (LMP – 180 days to LMP – 90 days) in addition to during spermatogenesis. This restriction was meant to ensure the father had no medication available that could be used during spermatogenesis, which would result in exposure misclassification.

^d^ Exposure contrasts: metformin continuation was defined as metformin monotherapy during spermatogenesis and 1 or more metformin prescription fills, but not for other antidiabetics before spermatogenesis (LMP – 150 days to LMP – 90 days), and 2 or more metformin prescription fills (LMP – 330 days to LMP – 150 days). Metformin discontinuation was defined as no antidiabetics during spermatogenesis and 1 or more metformin prescription fills, but not for other antidiabetics before spermatogenesis (LMP – 150 days to LMP – 90 days), and 2 or more metformin prescription fills (LMP – 330 days to LMP – 150 days). The requirement of 1 or more metformin prescription fills before spermatogenesis (LMP – 150 days to LMP – 90 days) and 2 or more metformin prescription fills during (LMP – 330 days to LMP – 150 days) was to ensure evidence of continuous metformin treatment prior to spermatogenesis.

Data analysis was performed from January 2023 to August 2024 using SAS, version 9.4 (SAS Institute). Additional details are provided in eMethods in Supplement 1.

## Results

The cohort included 780 637 linked pregnancies (mean [SD] age, fathers: 33.39 [4.70] years; mothers: 31.57 [4.24] years), with 3367 fathers exposed to metformin during spermatogenesis, 3763 to insulin, 1331 to sulfonylureas, and 1675 to other antidiabetic mediations (groups not mutually exclusive).

The prevalence of MCM^3^ in offspring with unexposed fathers was 3.79% (95% CI, 3.75%-3.83%), which is higher than the prevalence in offspring with antidiabetic-exposed fathers (Figure). However, the prevalence of MCM for metformin exposure was not higher during spermatogenesis than before or after spermatogenesis: 4.83% (95% CI, 4.12%-5.63%) before, 4.87% (95% CI, 4.18%-5.64%) during, and 4.91% (95% CI, 4.30%-5.58%) after spermatogenesis. Of note, 3157 fathers (93.8%) with metformin exposure during spermatogenesis were also exposed before and/or after spermatogenesis. The adjusted RR comparing metformin use with no metformin use was 1.02 (95% CI, 0.76-1.36) during spermatogenesis; 1.16 (95% CI, 0.89-1.52) before spermatogenesis; and 0.88 (95% CI, 0.66-1.17; last menstrual period [LMP] to LMP + 90 days) and 1.16 (95% CI, 0.90-1.48; LMP + 90 to LMP + 180 days) after spermatogenesis.

**Figure.**
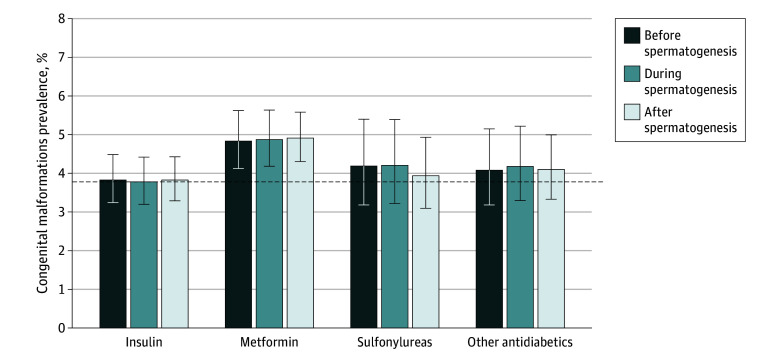
Prevalence of Major Congenital Malformations After Paternal Exposure to Antidiabetic Medications The dashed horizontal line represents the risk for pregnancies without paternal exposure to antidiabetic medications between last menstrual period (LMP) – 180 days and LMP + 180 days. Before spermatogenesis indicates LMP – 180 days to LMP – 90 days. During spermatogenesis indicates LMP – 90 days to LMP. After spermatogenesis indicates LMP to LMP + 180 days. Error bars represent the mid-*P* value CIs. Cohort inclusion criteria were paternal continuous insurance enrollment from LMP – 180 days through LMP + 180 days; maternal continuous insurance enrollment from LMP – 180 days through delivery + 90 days; infant continuous insurance enrollment through delivery + 90 days, unless the infant died sooner. Exclusion criteria were births with maternal diagnoses of diabetes or hypertension and births with maternal use of diabetes or cardiovascular medications.

The different exposure contrasts in the second study design did not provide robust evidence of an increased risk associated with metformin. Elevated RRs observed in the nonuser comparison were attenuated in both the active comparator and discontinuation analyses (Table). No genital defects pattern was observed.

## Discussion

While a small increased risk associated with paternal metformin exposure cannot be excluded, the association was not specific to the spermatogenesis period and is likely attributable to residual confounding by cardiometabolic risk factors. Study limitations include the potential for misclassification and residual confounding. Additionally, inclusion of only commercially insured pregnancies may limit representation of the entire obstetric population.

Two cohort studies did not confirm the initial signal reported for paternal preconception metformin use (RR, 1.40; 95% CI, 1.08-1.82) in the Danish study and its subsequent re-analysis.^1,4^ In an Israeli birth cohort, the authors reported an adjusted RR of 1.00 (95% CI, 0.76-1.31) for metformin overall.^5^ A cross-national study in Norway and Taiwan reported a pooled adjusted RR of 0.89 (95% CI, 0.77-1.03) compared with no metformin use among men with type 2 diabetes.^6^ Neither study found an association with genital or other organ-specific malformations. The accumulated evidence thus far does not support the initial report from the Danish study, suggesting this concern should not factor into metformin treatment decisions.

## Notes

Supplement 1.
eMethods.

eReferences

Supplement 2.
Data Sharing Statement
