# Supplementary material for: Paternal Preconception Metformin Use and Offspring Risk of Congenital Malformations
Source: JAMA Netw Open. 2025 Jun 12;8(6):e2515002. doi: 10.1001/jamanetworkopen.2025.15002 (PMC12163650; doi:10.1001/jamanetworkopen.2025.15002)
Supplement: Supplement 1. — eMethods. eReferences [file jamanetwopen-e2515002-s001.pdf]

Supplemental Online Content

Huybrechts KF, Straub L, Rotem RS, Bateman BT, Hernandez-Diaz S. Paternal preconception metformin use and offspring risk of congenital malformations. *JAMA Netw Open*. 2025;8(6):e2515002. doi:10.1001/jamanetworkopen.2025.15002

eMethods

eReferences

This supplemental material has been provided by the authors to give readers additional information about their work.

© 2025 Huybrechts KF et al. *JAMA Network Open*

eMethods

Study Cohort

The development of the mother-infant linked cohort has been described in detail before.[1,2] Briefly, we identified all completed pregnancies in women and adolescents 12 to 55 years of age and linked these pregnancies to liveborn infants. Using a validated algorithm, we estimated the date of the last menstrual period on the basis of the delivery date and diagnostic codes indicative of preterm delivery.[3] Fathers were linked by matching the first 10 digits of the family ID, indicating that individuals are on the same insurance plan, and as such are likely to be in the same family unit. Additional details regarding the father linkage are described in McEwan et al.[4] We required maternal continuous insurance enrollment from LMP-180 days through delivery+90 days; infant enrollment through delivery+90 days, unless they died sooner; and paternal enrollment from LMP-180 days through LMP+180 days.

The data source includes rich patient-level information on demographics (e.g., age), insurance enrollment, outpatient medication dispensings, outpatient and emergency department visits and hospitalizations, as well as their accompanying diagnoses and procedures (e.g., delivery). The pregnancy cohort has previously been used in multiple studies evaluating the safety of medications during pregnancy.[e.g.,5-7]

Exposure Contrasts

In the second study approach which aimed to disentangle associations with metformin use from those with familial cardiometabolic conditions, six different exposure contrasts were considered. The first two scenarios compared metformin – as monotherapy (scenario #1) and as mono- or combination therapy (scenario #2) – to no antidiabetic treatment. Since the potential for confounding is high in such a non-user comparison, we also considered active comparator scenarios. In defining these scenarios, we accounted for the fact that different treatments are indicated at different stages in disease progression.[8] We compared metformin to other antidiabetic treatments (Scenario #3), recognizing that metformin is frequently used as a first line treatment and there may be residual confounding by disease severity. Since insulin is frequently alone or in combination used when other treatments fail, we compared "metformin + insulin" versus "insulin alone" (scenario #4). Finally, we compared metformin continuation – as monotherapy (scenario #5) and as mono- or combination therapy (scenario #6) – to metformin discontinuation.

Major Congenital Malformations

Major congenital malformations were identified with algorithms that use inpatient or outpatient claims in infant records within 90 days after birth or in maternal records within 30 days after delivery. Outcome algorithms and corresponding positive predictive values are provided in the table below.[9,10,11] Briefly, we required at least two dates with a malformation diagnosis code, at least one date with a diagnosis code and a procedure code related to repair, or at least one date with a diagnosis code and infant death. An infant was considered to have a major congenital malformation if there was a diagnosis recorded for any of the 14 organ-specific malformations.

© 2025 Huybrechts KF et al. *JAMA Network Open*

Malformation Group	Malformation Subgroup	ICD-9 Dx	ICD-10 Dx	Citation or validation results
Cardiac	Conotruncal Defects	745.0x, 745.1x, 745.2x	Q20.0-Q20.3, Q20.5, Q20.8, Q21.3	PPV for cardiac malformations overall: 77.6% (65.7%-86.2%)<br>Source: Palmsten 2014<br><br>PPV for ventricular septal defect: 100% (91%-100%)<br>Source: Bateman 2021<br><br>PPV for ASD: 100% (91%-100%)<br>Source: Bateman 2021
	Single Ventricle	745.3x	Q20.4	
	Ventricular Septal Defect	745.4x	Q21.0	
	ASD	745.5x AND no preterm	Q21.1 AND no preterm	
	AV Septal Defect	745.6x	Q21.2	
	Right sided defects	746.00, 746.01, 746.09, 746.1x, 746.2x, 746.83, 747.3x AND no preterm, 746.02 AND no preterm	Q22, Q22.0, Q22.1 AND no preterm, Q22.3-Q22.9, Q24.3, Q25.5 AND no preterm, Q25.6 AND no preterm, Q25.7x AND no preterm	
	Left sided defects	747.1x, 747.2x, 746.3x, 746.5x, 746.7x, 746.81, 746.82	Q23, Q23.0, Q23.2, Q23.4, Q23.8, Q23.9, Q24.2, Q24.4, Q25.1x-Q25.4x	
	PDA	747.0x and no preterm	Q25.0 AND no preterm	
	PPHN	(416.0x or 747.83) and no preterm	(I27.0, P29.3x) AND no preterm	
	Great cardiac veins	747.4, 747.41, 747.42	Q26.0-Q26.4	
	Other cardiac	745.7x,745.8x, 746.8, 746.84-746.89	Q20.6, Q21.4, Q21.8, Q24.0, Q24.1, Q24.5, Q24.6, Q24.8, Q25.8	
	Cardiac NOS	745[1], 745.9, 746[1], 746.9x (do not count 746.99), 747	Q20, Q20.9, Q21, Q21.9, Q24, Q24.9, Q25, Q25.9	
Oral cleft		749.xx	Q35.x-Q37.x	PPV for non-cardiac malformations overall: 86% (74%-94%)<br>Source: He 2020<br><br>PPV for oral cleft: 96% (85%-99%)<br>Source: Bateman 2021
Central Nervous System		740.xx-742.xx	Q00.x-Q07.x	
Eye		743.xx (do not count 743.6x, 743.8x)	Q10[1], Q10.4, Q10.7, Q11.x-Q15.x (do not count Q13.5)	

© 2025 Huybrechts KF et al. *JAMA Network Open*

Malformation Group	Malformation Subgroup	ICD-9 Dx	ICD-10 Dx	Citation or validation results
Ear		744.0x, 744.23, 744.3x	Q16.x-Q17.x (do not count Q17.0, Q17.3, Q17.5)	
Other vascular (non-cardiac)		747.40, 747.49, 747.6x-747.9x (do not count 747.83)	Q26.5-Q26.9, Q27.x-Q28.x (do not count Q27.0, Q27.4)	
Respiratory		748.xx (do not count 748.1x, 748.3x)	Q30.0, Q30.1, Q31.0, Q32.x (do not count Q32.0), Q33.x (do not count Q33.1), Q34.x	
Gastrointestinal		750.xx-751.xx (do not count 750.0x, 750.1x, 751.0x)	Q38.x-Q45.x (do not count Q38.1, Q38.2, Q38.3, Q43.0)	
Genital (male and female)		752.xx (do not count 752.42, 752.52) (in addition, do not count 752.5x if preterm), 756.71	Q50.xx-Q52.xx (do not count Q52.3, Q52.5), Q53.0x, Q53.1xx AND no preterm, Q53.2xx AND no preterm, Q53.9 AND no preterm, Q54.xx-Q56.xx (do not count Q55.22), Q64.0, Q79.4	
Urinary		753.xx (do not count 753.7x), 756.71	Q60.xx-Q64.xx (do not count Q64.0, Q64.4), Q79.4	
Musculoskeletal		754.1x, 754.2x, 756.xx (do not count 756.2x, 756.7x)	Q68.0, Q75.x-Q78.x (do not count Q76.5), Q79.0, Q79.1, Q79.6, Q79.8, Q79.9	
Limb defects		755.xx (do not count 755.65, 755.63), 754.4x-754.8x (do not count 754.81, 754.82)	Q65.81, Q65.82, Q66.xx, Q68.1-Q68.8, Q69.x-Q74.x	
Other		757.0x, 757.1x, 759.xx (do not count 759.5x, 759.81-759.83)	Q80.8, Q80.9, Q86.x, Q89.xx	

© 2025 Huybrechts KF et al. *JAMA Network Open*

eReferences

1. Palmsten K, Huybrechts KF, Mogun H, et al. Harnessing the Medicaid Analytic eXtract (MAX) to Evaluate Medications in Pregnancy: Design Considerations. PLoS One 2013;8(6):e67405. (In Eng). DOI: 10.1371/journal.pone.0067405.

2. MacDonald SC, Cohen JM, Panchaud A, McElrath TF, Huybrechts KF, Hernandez-Diaz S. Identifying pregnancies in insurance claims data: Methods and application to retinoid teratogenic surveillance. Pharmacoepidemiol Drug Saf 2019;28(9):1211-1221. DOI: 10.1002/pds.4794.

3. Zhu Y, Thai TN, Hernandez-Diaz S, et al. Development and Validation of Algorithms to Estimate Live Birth Gestational Age in Medicaid Analytic eXtract Data. Epidemiology 2023;34(1):69-79. DOI: 10.1097/EDE.0000000000001559.

4. McEwen I, Huybrechts KF, Straub L, Hernandez-Diaz S. Patterns of paternal medication dispensation around the time of conception. Paediatr Perinat Epidemiol 2024;38(6):461-466. DOI: 10.1111/ppe.13098.

5. Hernandez-Diaz S, Straub L, Bateman BT, et al. Risk of Autism after Prenatal Topiramate, Valproate, or Lamotrigine Exposure. N Engl J Med 2024;390(12):1069-1079. DOI: 10.1056/NEJMoa2309359.

6. Straub L, Hernandez-Diaz S, Bateman BT, et al. Association of Antipsychotic Drug Exposure in Pregnancy With Risk of Neurodevelopmental Disorders: A National Birth Cohort Study. JAMA Intern Med 2022;182(5):522-533. DOI: 10.1001/jamainternmed.2022.0375.

7. Yland JJ, Huybrechts KF, Wesselink AK, et al. Perinatal Outcomes Associated With Metformin Use During Pregnancy in Women With Pregestational Type 2 Diabetes Mellitus. Diabetes Care 2024;47(9):1688-1695. DOI: 10.2337/dc23-2056.

8. ElSayed NA, Aleppo G, Aroda VR, et al. 9. Pharmacologic Approaches to Glycemic Treatment: Standards of Care in Diabetes-2023. Diabetes Care 2023;46(Suppl 1):S140-S157. DOI: 10.2337/dc23-S009

9. Palmsten K, Huybrechts KF, Kowal MK, Mogun H, Hernández-Díaz S. Validity of maternal and infant outcomes within nationwide Medicaid data. Pharmacoepidemiol Drug Saf. 2014 Jun;23(6):646-55. doi: 10.1002/pds.3627. Epub 2014 Apr 16. PMID: 24740606; PMCID: PMC4205050.

10. Bateman BT, Hernandez-Diaz S, Straub L, Zhu Y, Gray KJ, Desai RJ, Mogun H, Gautam N, Huybrechts KF. Association of first trimester prescription opioid use with congenital

© 2025 Huybrechts KF et al. *JAMA Network Open*

malformations in the offspring: population based cohort study. BMJ. 2021 Feb 10;372:n102. doi: 10.1136/bmj.n102. PMID: 33568363; PMCID: PMC7873721.

11. He M, Huybrechts KF, Dejene SZ, Straub L, Bartels D, Burns S, Combs DJ, Cottral J, Gray KJ, Manning-Geist BL, Mogun H, Reimers RM, Hernandez-Diaz S, Bateman BT. Validation of algorithms to identify adverse perinatal outcomes in the Medicaid Analytic Extract database. Pharmacoepidemiol Drug Saf. 2020 Apr;29(4):419-426. doi: 10.1002/pds.4967. Epub 2020 Mar 2. PMID: 32124511.

© 2025 Huybrechts KF et al. *JAMA Network Open*
