# Supplementary material for: Paternal Preconception Metformin Use and Offspring Risk of Congenital Malformations
Source: JAMA Netw Open. 2025 Jun 12;8(6):e2515002. doi: 10.1001/jamanetworkopen.2025.15002 (PMC12163650; doi:10.1001/jamanetworkopen.2025.15002)
Supplement: Supplement 2. — Data Sharing Statement [file jamanetwopen-e2515002-s002.pdf]

Data Sharing Statement

Huybrechts. Paternal Preconception Metformin Use and Offspring Risk of Congenital Malformations. *JAMA Netw Open*. Published June 12, 2025.
doi:10.1001/jamanetworkopen.2025.15002

Data
Data available: No

Additional Information
Explanation for why data not available: Authors are not allowed to share the data due to Data Use Agreements. However, the data are publicly available and can be requested from the data holders.
